# Three-Dimensional Imaging of the Mouse Neurovasculature with Magnetic Resonance Microscopy

**DOI:** 10.1371/journal.pone.0022643

**Published:** 2011-07-27

**Authors:** Arvind P. Pathak, Eugene Kim, Jiangyang Zhang, Melina V. Jones

**Affiliations:** 1 Russell H. Morgan Department of Radiology and Radiological Science, The Johns Hopkins University School of Medicine, Baltimore, Maryland, United States of America; 2 Sidney Kimmel Comprehensive Cancer Center, The Johns Hopkins University School of Medicine, Baltimore, Maryland, United States of America; 3 Department of Biomedical Engineering, The Johns Hopkins University School of Medicine, Baltimore, Maryland, United States of America; 4 Department of Neurology, The Johns Hopkins University School of Medicine, Baltimore, Maryland, United States of America; National Institute of Health, United States of America

## Abstract

Knowledge of the three-dimensional (3D) architecture of blood vessels in the brain is crucial because the progression of various neuropathologies ranging from Alzheimer's disease to brain tumors involves anomalous blood vessels. The challenges in obtaining such data from patients, in conjunction with development of mouse models of neuropathology, have made the murine brain indispensable for investigating disease induced neurovascular changes. Here we describe a novel method for “whole brain” 3D mapping of murine neurovasculature using magnetic resonance microscopy (μMRI). This approach preserves the vascular and white matter tract architecture, and can be combined with complementary MRI contrast mechanisms such as diffusion tensor imaging (DTI) to examine the interplay between the vasculature and white matter reorganization that often characterizes neuropathologies. Following validation with micro computed tomography (μCT) and optical microscopy, we demonstrate the utility of this method by: (i) combined 3D imaging of angiogenesis and white matter reorganization in both, invasive and non-invasive brain tumor models; (ii) characterizing the morphological heterogeneity of the vascular phenotype in the murine brain; and (iii) conducting “multi-scale” imaging of brain tumor angiogenesis, wherein we directly compared in vivo MRI blood volume measurements with ex vivo vasculature data.

## Introduction

The neurovasculature plays a critical role in a wide range of neuropathological processes from dementia to stroke to tumors [1]. In addition, the physiological underpinnings of image contrast in functional MRI (fMRI) critically involves the neurovasculature [2], and is central to understanding drug delivery and pharmacokinetics of novel therapies in the brain [3], [4]. The challenges in obtaining high-resolution vascular data from patients, combined with new pre-clinical models of neuropathology, have made the mouse brain indispensable to investigations of these areas [5], [6].

Historically, high-resolution characterization of the murine neurovascular architecture has involved either two-dimensional (2D) measurements made on tissue sections from 3D specimens [7] or three-dimensional (3D) measurements made on corrosion casts [8]. Although 3D reconstruction of 2D tissue slices generates high resolution imaging data, the practical limit as to the number of adjacent tissue slices that can be obtained limits the region of brain that can be interrogated. Additionally, recovering the 3D blood vessel and white matter fiber geometry once destroyed by tissue sectioning requires complex reconstruction techniques. Conversely, corrosion casts allow extensive brain regions to be analyzed but require dissolving of tissues to visualize cast vessels, and thus precludes simultaneous assessment of the vasculature and neuroanatomy.

Recent advances in imaging technology have permitted the visualization of the murine neuroarchitecture in exquisite detail using magnetic resonance microscopy (μMRI), a type of 3D MRI that offers high *ex vivo* imaging resolutions (currently ∼20–30 µm^3^) [9]. Using μMRI one can obtain images with different kinds of “physiological stains” or image contrast mechanisms, e.g. diffusion tensor imaging (DTI) to visualize the orientations of white matter fibers in the brain [10].

Studies describing wide-area-mapping of the murine brain vasculature with 3D μMRI have been limited due to the difficulties associated with sample preparation, contrast mechanism employed, image co-registration, and image processing for vessel extraction. For example, Dorr et al combined vascular data obtained using micro computed tomography (μCT) with a μMRI murine brain atlas. While this approach exploits the strengths of each modality, co-registration of these 3D image volumes is challenging [11]. This is especially true when it involves registration with DTI or the abnormal vasculature of brain tumors. Gelatin doped with gadolinium chelates has also been employed to image both, mouse embryo [12] and adult rat brain [13] vasculature using μMRI. Unfortunately, the use of gelatin perfusate is not ideal as it needs to be mixed with high concentrations of gadolinium chelate to achieve sufficient T_1_ relaxation, and diffusion of these chelates out of the blood vessels over time results in loss of vascular contrast. Here we describe a new method for extending the utility of 3D μMRI to include “whole brain” mapping of the vasculature that circumvents the abovementioned drawbacks. We validated our vascular μMRI technique using optical and μCT techniques and present three important biological applications to demonstrate the utility of this approach for characterizing changes in the neurovascular microenvironment wrought by invasive and non-invasive pre-clinical brain tumor models.

To circumvent the limitations of traditional vascular imaging approaches, we perfused murine brains with an inert silicone rubber compound (MICROFIL®, FlowTech Inc., MA) which has a low viscosity that permits complete filling of the murine cerebral vasculature [14]. Following polymerization, Microfilled blood vessels appear dark on gradient-echo MRI images due to the absence of mobile water and potential susceptibility contrast. Being hydrophobic restricts the Microfil® to the vasculature with minimal extravasation. Additionally, because Microfil® is radio-opaque and light microscopy opaque, it enables validation of the μMRI-derived vascular data with μCT, x-ray imaging and bright field whole mount microscopy as we have demonstrated in this study.

We demonstrate the potential of our μMRI technique via several applications that include combined 3D imaging of angiogenesis-induced vascular remodeling and white matter reorganization in both, invasive and non-invasive brain tumor models; characterization of the morphological heterogeneity of the neurovasculature; and “multi-scale” imaging of brain tumor angiogenesis. Since μMRI generates digitized 3D images, the neurovascular data derived from it can be employed in a plethora of applications ranging from the study of cerebral hemodynamics [15] to image contrast mechanisms in MRI [16].

## Results

### Validation of μMRI-derived brain vasculature with μCT

**Figure 1:**
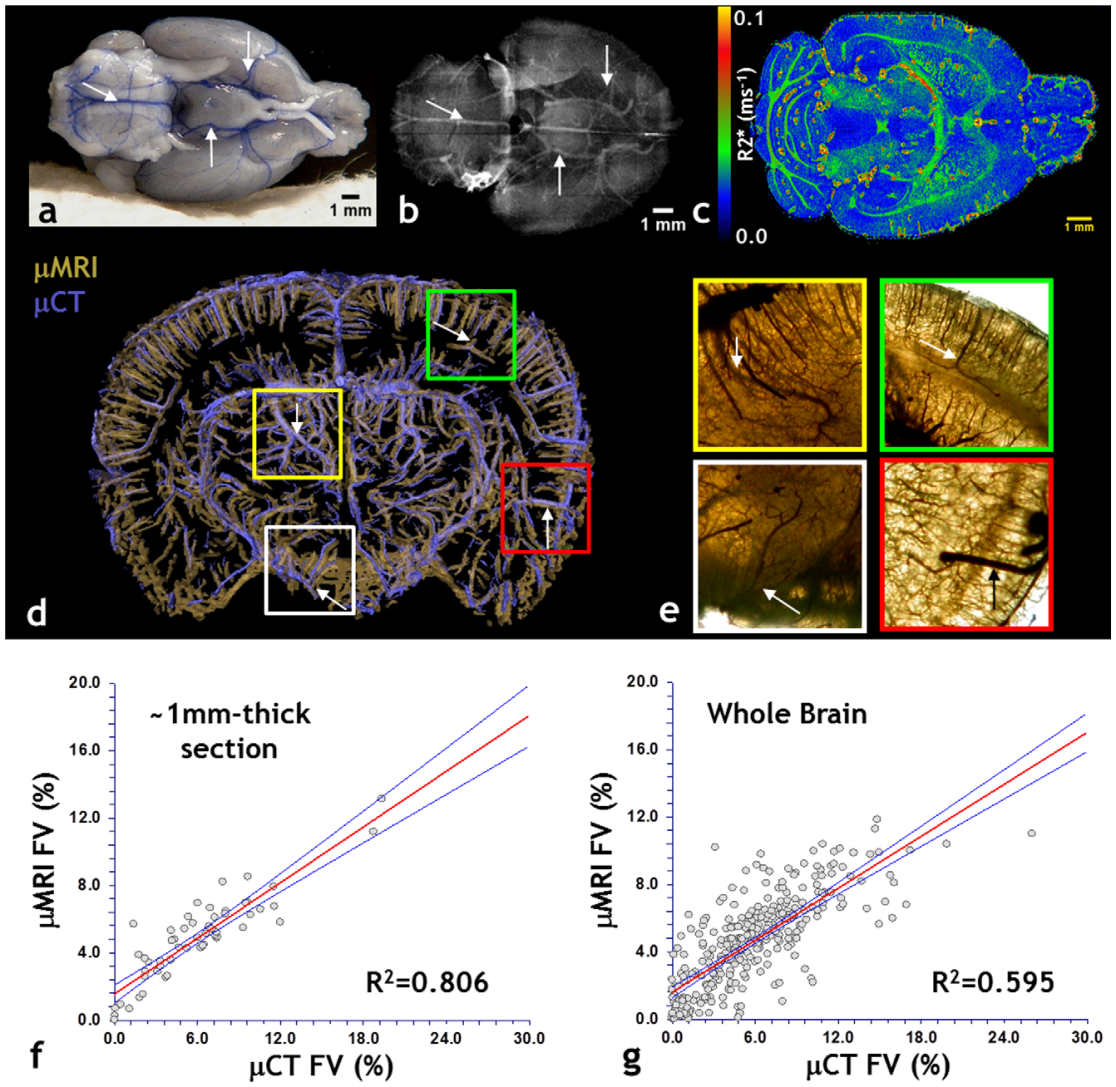
3D imaging of the murine neurovasculature with μMRI and validation with μCT and optical microscopy. (a) Photograph of a freshly excised mouse brain showing blue Microfil® perfused vessels (arrows). (b) X-ray radiograph of the same brain in which radio-opaque microfilled vessels are clearly visible. Arrows indicate major vessels that are also visible in (a). (c) Slice through the 3D R_2_* map of the same brain. The Microfil-brain tissue interface is characterized by elevated R2* (hot colors) values. Note that background voxels are assigned R2* of zero. (d) ∼1.2 mm slab from another intact brain, in which μMRI-derived vasculature (gold) is overlaid on that acquired using μCT (purple). One can clearly visualize the vascular architecture and the agreement between μMRI and μCT. (e) Bright-field images (2×) of ROIs corresponding to colored squares in (d). Images are from a 1 mm thick, unstained brain section. Dark microfilled vessels provide corroboration of the μMRI data in (d). Arrows indicate major vessels that are also visible in (d). μCT data were resampled to match the μMRI spatial resolution, and the fractional vascular volume (FV) computed within 8×8×1 subvolumes for each dataset. The correlation between the μMRI and μCT-derived FVs for the 1 mm thick slice is plotted in (f). A similar analysis was conducted for the *whole* brain, wherein the FV was computed within 8×8×8 subvolumes for each dataset. The correlation between the μMRI and μCT-derived FVs for the *whole* brain is plotted in (g) and demonstrate good agreement between μMRI and μCT-derived vasculature. The red lines in (f) and (g) are the best linear fit to the data, and blue lines indicate the 95% confidence limits about the mean.

One of the principle ideas underlying vascular μMRI is to acquire a 3D representation of the murine cerebral vasculature at high spatial resolution while achieving “whole brain” spatial coverage. Brains were perfused with the silicone rubber compound Microfil® that is both, optically (**Fig. 1a**) and x-ray (**Fig. 1b**) opaque. The difference in magnetic susceptibility between the Microfilled vessels and surrounding cerebral tissue enhances the gradient-echo relaxation rate at the vessel boundaries as shown in **Fig. 1c**. Additionally, the vessel lumen appears dark due to the lack of mobile protons within the polymerized Microfil®. We validated the fidelity of the μMRI-derived vasculature by comparing it to that acquired using μCT and whole-mount optical microscopy. This was achieved by imaging the same brain sample using all three modalities. **Fig. 1d** demonstrates that there was excellent overlap between the co-registered μMRI and μCT-derived vasculatures. Additionally, bright field microscopy of a whole-mount brain section confirmed that the μMRI-visible features were Microfil®-bearing vessels (**Fig. 1e**). Finally, we confirmed the high quality of the μMRI data by assessing the correlation between the fractional blood volume (FV) computed from μMRI with that computed from μCT. We found that in spite of being computed from two different imaging modalities, there was strong correlation between FV values for both, a 1 mm thick brain slice (**Fig. 1f**), as well as over the entire brain sample (**Fig. 1g**). These data establish the fidelity of the μMRI-derived vasculature.

### Simultaneous visualization of brain tumor angiogenesis and invasion

**Figure 2:**
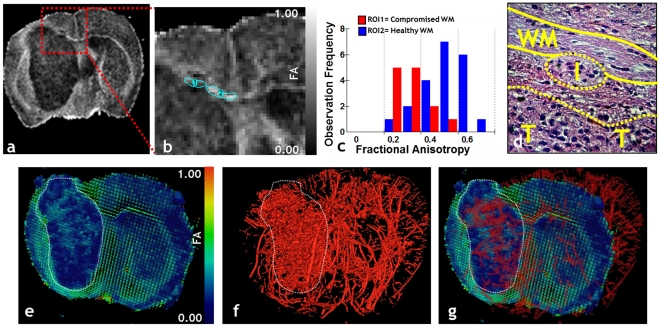
Simultaneous imaging of brain tumor angiogenesis and invasion with μMRI. (a) FA map of a patient-derived, invasive primary glioma model. (b) Zoomed view of the hatched region in (a) showing two ROIs in the corpus callosum for which the FA was analyzed. (c) Histograms of the FA from the ROIs in (b), wherein one can see that the FAs from ROI-1 are shifted toward lower values than those from ROI-2. (d) Histology (H&E) from the same region as in (b) in which one can see the white matter tract (WM) being infiltrated by a tuft of tumor cells (I). The tumor margin (T) is also visible in (d). (e) Visualization of the DTI tensors as 3D ellipsoid glyphs for one μMRI slice, wherein each ellipsoid is scaled according the values of the three principal eigen-vectors and color coded according to the FA. The invasive primary tumor (hatched outline) is identifiable by its lower FA in contrast to the contralateral brain. (f) Visualization of the 3D vasculature for the whole brain. Tumor vasculature (hatched outline) is dense and chaotic relative to that of the contralateral brain. (g) The image in (e) overlaid with that in (f) allows us to simultaneously assess the interaction between brain tumor angiogenesis and the effects of tumor invasion on the integrity of white matter tracts. The tumor ROI is highlighted by a hatched outline.

A principal advantage of vascular μMRI is the ability to combine 3D cerebral vasculature data with co-registered MRI data acquired using a range of complementary contrast mechanisms. **Fig. 2** illustrates the power of this approach for illuminating changes in the neuroarchitecture that accompany angiogenesis in an orthotopic, invasive human brain tumor model. For instance, the fractional anisotropy (FA) map (**Fig. 2a**), a scalar measure of the degree of anisotropy in a given imaging voxel, shows a dark area in the corpus callosum (**Fig. 2b**) in which the FA is lower than that of adjacent white matter (**Fig. 2c**). The histology (**Fig. 2d**) demonstrates that this lowering of FA correlates with invasion of the white matter tract by infiltrating brain tumor cells. Another method of visualizing changes in the neuroarchitecture is illustrated in (**Fig. 2e**). Here the tensor that describes the 3D shape of diffusion is represented as 3D ellipsoid glyphs in which each ellipsoid is scaled according the values of the three principal eigen-vectors, and color coded according to the FA. The invasive primary tumor is easily identifiable by its low FA in contrast to the contralateral brain. The 3D vasculature for the whole brain is shown in (**Fig. 2f**) in which the dense, chaotic tumor vasculature is obvious relative to the regular, hierarchical vasculature of the contralateral brain. Finally, the interaction between brain tumor angiogenesis and the effects of tumor invasion on the integrity of white matter tracts can be simultaneously visualized by combining these 3D data as shown in (**Fig. 2g**). Therefore, an added advantage of using Microfil as an MRI contrast agent is that its presence does not have any deleterious effects on diffusion-weighted MRI contrast.

### Zonal analysis of μMRI-derived vasculature

**Figure 3:**
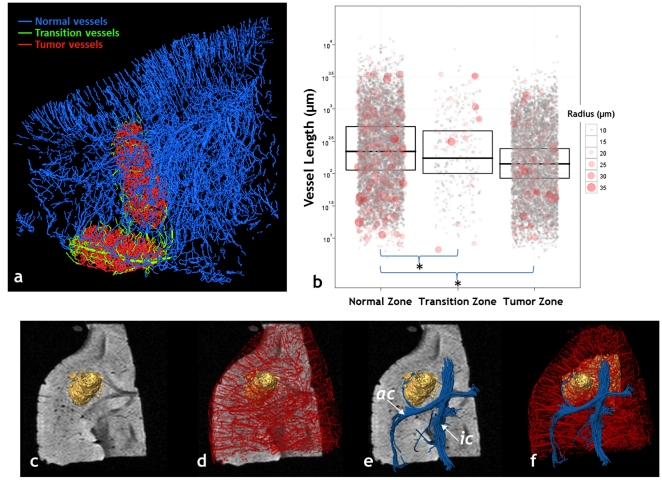
Ultra-high resolution 3D μMRI and “zonal” analyses of the neurovasculature. (a) 3D rendering of the neurovasculature in a non-invasive, 9L tumor bearing mouse brain acquired using ultra-high resolution (30 µm×30 µm×30 µm) μMRI. The vasculature has been color coded into three different “zones”: normal vessels (blue), tumor vessels (red) and vessels at the tumor-brain interface or transition zone (green). The transition-zone or tumor-brain tissue interface is crucial to understanding both, brain tumor angiogenesis and invasion. The radius and length of every individual vessel segment was measured in each zone. (b) Box plot of the average vessel length in each zone, wherein the width of each box includes 75% of the measured lengths and the median length is indicated by a horizontal line in each box. In addition, the radius of every vessel segment is plotted for each zone, with the color and size of each symbol proportional to the vessel radius. The normal zone exhibited significantly longer vessel segments compare to the transition (p = 0.002) and tumor zones (p<0.001), respectively. At this tumor stage, vessel radii were similar between the tumor and normal zones. These data demonstrate our ability to characterize the neurovasculature in physiologically relevant “zones”, and could provide new insight into the relationship between brain tumor angiogenesis and invasion. (c) T_2_-weighted μMRI slice through a 9L brain tumor (gold rendering) bearing brain. (d) 3D overlay of the neurovasculature acquired using ultra-high resolution μMRI. (e) 3D DTI image showing reorganization of the fibers of the anterior commissure (*ac*) and internal capsule (*ic*) around the tumor. (f) Overlay of (d) and (e) illustrating simultaneous changes in vascular and white matter structures.

The extensive spatial coverage of 3D μMRI permits one to sample the spatial heterogeneity of the underlying vascular phenotype. For example, it is well known that the vessel morphology of the angiogenic brain tumor is drastically different from that of the contralateral brain. Here we explicitly demonstrate the feasibility of characterizing differences in vessel segment length and radius in three different zones using “ultra-high” resolution μMRI (**Fig. 3a**). In addition to the *tumor* and *normal* zones, we defined a *transition* zone that straddles the tumor-brain tissue interface. We found that the normal zone had significantly longer vessel segments compare to the transition (p = 0.002) and tumor zones (p<0.001), and that vessel radii were heterogeneously distributed for this tumor stage (**Fig. 3b**). Ultra-high resolution μMRI permits the simultaneous assessment of vascularization and white matter reorganization in the presence of a non-invasive (9L) brain tumor model (**Fig. 3c–f**). In contrast to white matter tract infiltration seen in the invasive brain tumor model, the non-invasive 9L brain tumor simply displaces the surrounding white matter tracts (**Fig. 3e**).

### Multi-scale imaging of brain tumor angiogenesis

**Figure 4:**
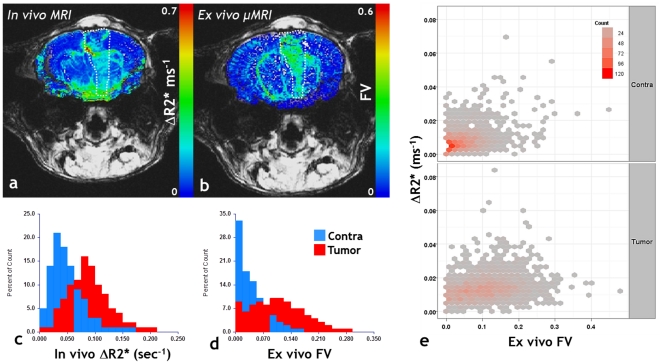
Bridging macroscale and microscale MRI. (a) In vivo macrovascular CBV (ΔR_2_*) map. (b) Co-registered *ex vivo* fractional blood volume (FV) map obtained from μMRI. The tumor ROI is highlighted by hatched lines in each panel and FV ranges from 0 to 1. (c) Histograms showing the relative distribution of the ΔR_2_* between tumor and contralateral ROIs. (d) Histograms showing the relative distribution of the FV between tumor and contralateral ROIs. Tumor blood volume is elevated relative to the contralateral brain across these “multi-scale” data. (e) 2D histograms of the macrovascular CBV measured *in vivo* versus the fractional blood volume assessed *ex vivo*. These data further demonstrate the utility of multi-scale imaging of brain tumor vascularization.

**Figure 5:**
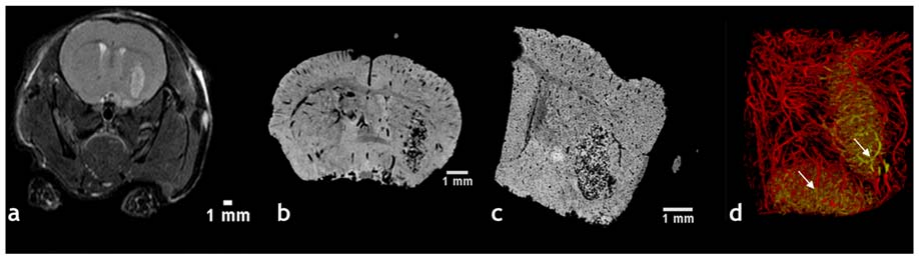
Multi-scale MRI of a 9L brain tumor model. (a) In vivo T_2_-weighted MRI at the ‘systemic’ scale (∼150 µm); ex vivo μMRI at two ‘intermediate’ scales: (b) ∼60 µm, and (c) ∼30 µm. (d) Ultra-high resolution vascular μMRI image in which vessels have been segmented into tumor vessels (gold) and normal vessels (red). One can clearly visualize the abnormal tumor vessel architecture and changes in vessel morphology at the tumor-host tissue interface (arrows).

The emergence of systems-biology approaches has necessitated the acquisition of “multi-scale” data to bridge the gap between various components of the biological system being studied [17]. Such data can be acquired using imaging methods and then be either incorporated into mathematical models or used to validate such models. Here we illustrate one instance of imaging angiogenesis in a 9L brain tumor bearing mouse brain at two spatial scales using entirely unique contrast mechanisms: *in vivo* using susceptibility-contrast arising from the superparamagnetic contrast agent Feridex (**Fig. 4a**), and *ex vivo* using contrast arising from the lack of free water protons in the Microfilled blood vessels (**Fig. 4b**). The former provides a map of the change in the gradient-echo relaxation rate (ΔR2*) which is reflective of the macrovascular or total cerebral blood volume (**Fig. 4a**), while the latter is the fractional volume (FV) map computed directly from the μMRI-derived vasculature (**Fig. 4b**). One can see that both, the *in vivo* MRI and *ex vivo* μMRI exhibit identical trends in blood volume, i.e., the tumor blood volume is elevated with respect to that in the contralateral brain (**Fig. 4c–d**). The 2D histogram (**Fig. 4e**) reiterates the trend in the blood volume measured using these independent methods and illustrates the correlation between them. **Fig. 5** illustrates the range of spatial resolutions that one can span, with *in vivo* MRI at one end, and ultra-high resolution μMRI at the other end of the spatial resolution spectrum.

## Discussion

Traditionally, the neurovasculature in murine models of disease has been characterized at two spatial scales: the “cellular” or microscopic scale using 3D optical techniques [18], [19], and the “systemic” or macroscopic scale using methods such as contrast enhanced MRI, magnetic resonance angiography (MRA) [20] [21] or computed tomography (CT) [11], [22] [17]. Optical approaches suffer from limited tissue penetration and provide relatively small fields of view. This makes “whole brain” coverage and co-registration with *in vivo* imaging data from complementary imaging modalities challenging. Conversely, μCT provides unparalleled 3D detail, but has limited soft tissue contrast (e.g. gray and white matter contrast), which is crucial to any comprehensive study of the neurovasculature. “Mesoscopic” resolution (20–40 µm) vascular μMRI bridges the gap between these spatial scales and enables imaging of the entire brain vasculature while simultaneously providing outstanding gray and white matter contrast.

While newer methods such as volumetric computed tomography (VCT) [23] and synchrotron tomography [24] are capable of vascular imaging at exquisite spatial resolutions, they lack soft tissue contrast and require specialized detectors and a high-energy synchrotron source, respectively. Compared to these methods, vascular μMRI has three major advantages. First, it does not require specialized MRI hardware, and is inexpensive and easy to implement on any μMRI scanner. Second, it provides exceptionally high quality, 3D images of the vasculature by exploiting a novel contrast mechanism that can be easily combined with other MRI contrast mechanism (e.g., DTI) without affecting their quality. Third, it yields stable samples that can be imaged non-destructively with MRI, CT or optical microscopy without requiring any additional contrast agents or labels.

A current limitation of using μMRI to image the vasculature of the whole mouse brain is that even at ∼30 µm resolution, arterioles, venules and capillary-sized vessels are undetectable. This difference is apparent when comparing the vascular coverage visible in μMRI (**Fig. 1d**) and that visible in μCT (**Fig. 1d**) and histology (**Fig. 1e**). This constraint on the μMRI spatial resolution results in partial volume effects that impact the calculation of morphological vascular parameters. For example, partial volume effects can lead to overestimations of vessel length, caliber and fractional volume, and an underestimation of vessel tortuosity.

With the availability of higher magnetic field imaging systems (9.4 Tesla or higher) and more powerful imaging gradients, it will be possible to acquire higher spatial resolution μMRI images. However, this improvement in resolution will come at the expense of increased image acquisition times, which will result in large size image data sets and limit the number of samples that can be imaged. While strategies such as doping the sample with gadolinium to shorten the tissue T_1_ or employing partial Fourier reconstruction schemes ameliorates protracted imaging times [25], achieving reasonably high signal-to-noise ratio (SNR) with smaller voxel sizes remains a technical challenge. This is further compounded by the fact that Microfilled vessels contain a limited amount of mobile “MR visible” water.

Both, excision from the skull and tissue fixation result in distortion of the brain tissue. For μMRI, the brain sample is enclosed in a 10mm glass NMR tube and for μCT the sample is embedded in a gel prior to imaging. Each of these steps also induce tissue distortions. Collectively, these tissue distortions preclude direct vessel-to-vessel co-registration between the μMRI and μCT data at their native spatial resolution. For example, at the ∼60 µm resolution of μMRI, misalignments of the order of a few μMRI voxels between the μMRI and μCT-derived vasculature precludes meaningful voxel-wise statistical analyses. Therefore, to validate the vascular coverage of the μMRI data with that derived from μCT, we computed the fractional blood volume (FV) for both datasets on a coarse spatial grid. We selected the FV as it is a morphological parameter that is function of both, vessel caliber and vessel density [26]. To ensure insensitivity to misalignments between the two datasets, we computed the FV by calculating the fractional occupancy of the binarized vasculature derived from the μMRI and μCT data within a coarse 8×8×8 spatial grid and found good agreement between the FVs obtained from the two methods. Due to the variety of measurement techniques employed, reported values for the blood volume in the mouse brain range from ∼0.5%–6% [22], [27], [28], [29], [30]. The FV of 3.2% computed from the μCT data fall within this range, while the FV of 7.9% computed from the μMRI data overestimates the FV due to partial volume effects. Although μMRI does not have the ability to resolve cortical capillaries, it is capable of “wide-area” or “whole brain” mapping of the neurovasculature. While it does offer superior coverage compared to most optical techniques, μMRI cannot outperform μCT in terms of spatial resolution. The advantage of μMRI is that one can employ complementary contrast mechanisms such as DTI, to simultaneously measure changes in the brain's microenvironment.

Recent evidence has emerged that antiangiogenic therapy in brain tumors promotes an invasive phenotype [31], [32]. This has created an urgent need for noninvasive methods to characterize the angiogenesis-invasion nexus and the efficacy of combined antiangiogenic/anti-invasive therapies in brain tumor models. Here, we demonstrated the feasibility of imaging changes in the whole brain microenvironment induced by both, invasive and non-invasive brain tumor models. While others have characterized brain tumor induced alterations in the brain's cytoarchitecture with μMRI [33], simultaneous imaging of the whole-brain vasculature has not been reported. It is this ability of μMRI to simultaneously visualize brain tumor vascularization and white matter remodeling over the entire murine brain that makes it a powerful new tool for understanding the interplay between angiogenesis and invasion in pre-clinical brain tumor models. In a recent study, we successfully demonstrated the ability of μMRI to phenotype global and zonal changes in the brain tumor microenvironment with tumor progression [34].

The evolution in our understanding of brain tumor angiogenesis has been the result of pioneering studies spanning the endothelial cell [35], microvasculature [36] and tissue level [37]. Many of these primary data on angiogenesis are in the form of images from pre-clinical models that provide a wealth of qualitative and quantitative information in many dimensions, and across different spatial scales. However, simultaneously visualizing changes in the complex angiogenic microenvironment at different spatial scales remains a challenge due to the lack of integration between micro- and macroscopic imaging data and the difficulty in obtaining such data from patients. Therefore, “mesoscopic” μMRI could be an expedient tool for integrating micro- and macroscopic angiogenesis imaging data. Here, we showed an example in which co-registered FV maps computed from *ex vivo* μMRI data were employed to validate *in vivo* macroscopic blood volume measurements. This imaging platform could easily be extended to establish a toolbox for integrating multi-scale data on brain tumor angiogenesis.

Our approach for μMRI imaging of the neurovasculature has several widespread applications: integration with extant MRI brain atlases, incorporation in biophysical models of MR contrast mechanisms, co-registration of *in vivo* imaging data with gene/protein data from immunohistochemistry, fluid dynamic simulations of tumor blood flow, multi-scale mathematical models of angiogenesis, and phenotyping pathological samples. The advantages and ease-of-implementation of vascular μMRI make it an indispensable tool for the study of neuropathologies that involve an aberrant neurovasculature.

## Methods

### Ethics Statement

All animals were handled in accordance with good animal practice as defined by the relevant national and/or local animal welfare bodies, and all animal work was conducted under a protocol (#MO07M287) approved by the Institutional Animal Care and Use Committee (IACUC) of Johns Hopkins University. The Johns Hopkins University animal facility is accredited by the American Association for the Accreditation of Laboratory Animal Care and meets National Institute of Health standards as set forth in the “Guide for the Care and Use of Laboratory Animals” (DHHS Publication No. (NIH) 85–23, Revised 1985).

### In Vivo MRI Protocol

9L and human primary brain tumor derived cells were orthotopically inoculated according to the protocol described in [26]. 9L brain tumor bearing animals were imaged *in vivo* on a 400 MHz Bruker spectrometer using the following sequences and parameters: (i) T_2_*-weighted (T_2_*w) multi-echo gradient echo (MGE), eight echoes, first TE = 5.0 ms, echo spacing = 5.0 ms, TR = 800 ms, NA = 12. (ii) T_2_w rapid acquisition with refocused echoes (RARE), six echoes, first TE = 12.0 ms, echo spacing = 12.0 ms, TR = 2000 ms, NA = 4. For all scans: in-plane resolution = 0.1 mm×0.1 mm, 16 coronal slices, slice thickness = 1 mm. Animals were imaged under isoflurane anesthesia, with their body temperature maintained at 37°C using a heating blanket. Images were acquired before and after equilibration of the superparamagnetic MRI contrast agent Feridex (AMAG Pharmaceuticals, Inc., Cambridge, MA), administered at a dose of 3.73mg/ml of Fe via a tail vein cannula. Pre- and post-contrast R_2_ (i.e. 1/T2) and R_2_* (i.e. 1/T_2_*) maps were calculated from the *in vivo* T_2_w and T_2_*w images, respectively.

### Sample Preparation for ex vivo μMRI and μCT imaging

9L and primary brain tumor-bearing animals were sacrificed approximately two and five weeks post-inoculation, respectively. Brains were perfusion fixed, followed by perfusion with a silicone rubber compound called Microfil® (FlowTech Inc., MA) according to a method we previously developed [38]. Briefly, mice were deeply anesthetized with isoflurane then perfused via the left ventricle – first with heparinized PBS, then with 10% buffered formalin for fixation, and finally with a 1∶2 mixture of Microfil® to diluent and 5% (v/v) curing agent. The Microfil® was allowed to cure at room temperature for 90 minutes, and then the heads were fixed in cold paraformaldehyde (PFA) for two days. Next, brains were excised from their skulls and stored in cold PFA. Twenty-four hours prior to imaging, each brain was immersed in PBS doped with 1 mM Gd-DTPA (GE Healthcare) to enhance soft tissue contrast. Each brain was imaged in an NMR tube filled with Fomblin® (Solvay Solexis, Milano, Italy), which provides a dark, uniform image background as it does not contribute any proton signal to the MR images.

### Magnetic Resonance Microscopy Protocol

Post-fixation, brain were imaged on a 400 MHz spectrometer using the following sequences and parameters: (i) T_2_*-weighted (T_2_*w) multi-echo gradient echo (MGE), FOV = 16mm×10mm×8mm, TE = 4.9/9.7/14.5/19.3 ms, TR = 150 ms, resolution = 62 µm×62 µm×63 µm, 14 averages. (ii) T_2_w rapid acquisition with refocused echoes (RARE), TE/TR = 63.4/2500 ms, resolution = 102 µm×102 µm×101 µm, 4 averages. (iii) Diffusion tensor imaging (DTI), TE/TR = 26.8/2000 ms, resolution = 94 µm×97 µm×600 µm, 6 averages; one non-diffusion weighted image and six diffusion weighted (DW) images were acquired with a b-value of 1700 s/mm^2^ and diffusion sensitizing gradient orientations: [1,1,0], [1,0,1], [0,1,1], [−1,1,0], [1, 0, −1], [0, −1, 1]. To enable zonal analysis of the vascular morphology, ultra-high resolution MGE μMRI data were acquired with a resolution = 33 µm×31 µm×30 µm. Along with the ultrahigh-resolution vascular μMRI, we also acquired co-registered DWI with a resolution = 66 µm×52 µm×44 µm, b-value = 1500 s/mm^2^ and the same diffusion sensitizing gradient orientations as before.

### Micro Computed Tomography Protocol

Samples were sent to Numira Biosciences (Salt Lake City, UT) and were imaged on a high-resolution, volumetric micro-CT scanner (μCT40, ScanCo Medical, Zurich, CH). The image data was acquired with the following parameters: 8 µm isotropic voxel resolution at 55 kVp 300 ms exposure time, 2000 views and 5 frames per view. The μCT generated DICOM files were then converted to a .raw file format for additional analysis.

### Image Processing

#### Calculation of in vivo relative blood volume maps

Pre- and post-contrast R_2_ (i.e. 1/T_2_) and R_2_* (i.e. 1/T_2_*) maps were calculated from the *in vivo* T_2_w and T_2_*w images, respectively, via voxel-wise monoexponential fitting of the intensity vs. time curves:
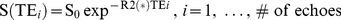
(1)Where S(TE*_i_*) is the measured signal intensity at the *i* ^th^ TE and S_0_ is the signal intensity at t = 0. The following constraints were imposed during curve-fitting: 0<S_0_<2^16^ (16-bit data) and 0<R_2_^(^*^)^<1 (T_2_>1 ms). Finally, the macrovascular or total (i.e. ΔR_2_*) cerebral blood volume maps were calculated:
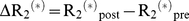
(2)The background of the R2*map was masked out and each background voxel assigned an R2* of zero. All images were processed using Analysis of Functional NeuroImages (AFNI) software [39].

#### Vessel segmentation

**Figure 6:**
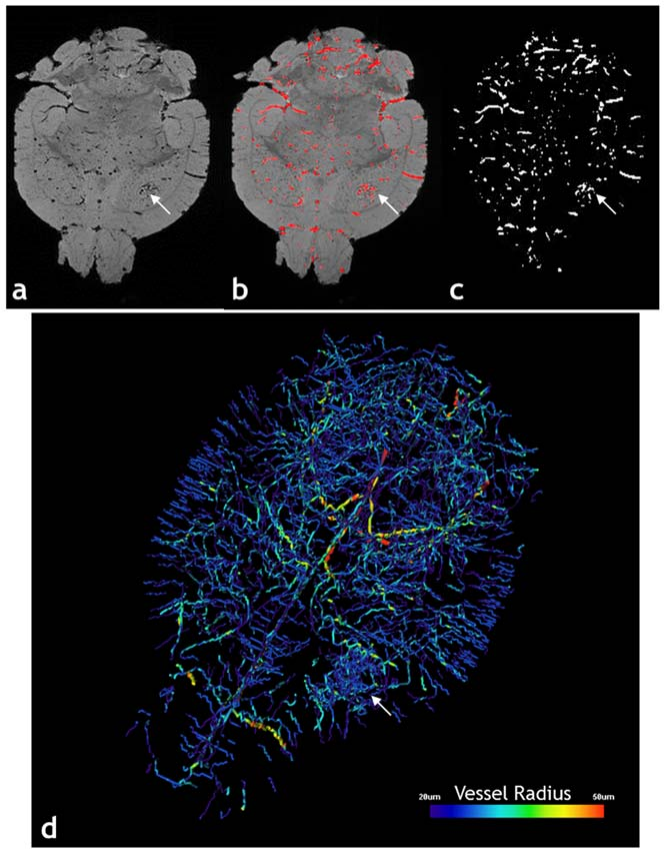
Segmentation of the Vasculature from μMRI Data. Image processing steps involved in the extraction of the 3D vasculature from the raw GE μMRI data for a 9L tumor (arrow in all panels) bearing mouse brain: (a) *Ex vivo* T_2_*-weighted image corresponding to the 1^st^ TE; (b) Blood vessels (red) segmented out using the “tubeness” filter overlaid on the raw data in (a). (c) Binarized vasculature obtained by thresholding the tubeness data in (b) followed by removal of isolated voxels. (d) Volume rendering of the μMRI-derived vasculature, color-coded by average vessel radius.

3D MGE images corresponding to the first TE, which have the highest SNR (signal-to-noise-ratio) and the least susceptibility related artifacts, were imported into ImageJ (Rasband, W.S., National Institutes of Health, Bethesda, Maryland, USA, http://rsb.info.nih.gov/ij/) for blood vessel extraction and computation of vascular morphological parameters. The image processing procedure for vessel extraction is summarized in **Fig. 6**. Briefly, the vasculature was automatically segmented from the brain using a Hessian-based, multi-scale “tubeness” filter [40]. This filter determines how “tube-like” a voxel is by convolving the image with a spherical Gaussian kernel with standard deviation *σ*, computing the Hessian matrix at each voxel, and computing a “tubeness” metric from the Hessian eigenvalues. The sensitivity of the “tubeness” filter to tubular structures of varying radii can be tuned by varying *σ*. Therefore, we implemented a multi-scale integration of filter responses using three *σ* values (*σ* = 0.8, 1.0, 1.2) to optimally extract vessels of all radii in the μMRI image [40]. Next, a binary 3D vascular structure was obtained by applying an iterative threshold algorithm [41] on the result of the “tubeness” filter. To eliminate small, disconnected objects and gaps in the vasculature, the binary image was subjected to a 3D size filter to remove isolated background and foreground regions smaller than three voxels.

A similar method was used to extract the vessels from the μCT data. Because of the higher spatial resolution, a wider range of *σ* values (*σ* = 1.5^n^, n = 0, 1,…,5) was used for the “tubeness” filter, and a threshold of 27 voxels was used for the 3D size filter.

#### Validation of μMRI with μCT and optical imaging

We validated the μMRI-derived vasculature with the μCT-derived vasculature. To do this, we used landmark-based registration to manually co-register the μMRI and μCT-derived whole brain vasculature in Amira (Visage Imaging Inc., CA). We then extracted ∼1mm-thick slabs from the whole brain μMRI and μCT data and subjected them to another landmark-based registration to refine the co-registration between them. For all the co-registrations, landmarks were placed on large vessel bifurcations visible in both datasets, and aligned using the thin-plate spline method [42]. The μCT data was then resampled to the μMRI resolution in AFNI to approximate the vascular structure that would be obtained from μMRI given the μCT vasculature as the ground truth. For both, μMRI and μCT datasets we calculated the fractional occupancies of vessels in an 8×8×8 (voxel size = 2mm×1.25mm×1mm) grid for the whole brain and an 8×8×1 grid for the 1mm-thick slab using AFNI. Voxel-wise correlations of the fractional blood volume maps were then computed between these two datasets. Finally, the μMRI and μCT data were also validated with optical microscopy by visual inspection. The 1 mm-thick slab used in the μMRI vs. μCT correlation analysis was excised and the tissue cleared by graded immersion in glycerin. The tissue was first immersed in a mixture of 50% glycerin and water, and every 24 hours, the glycerin concentration was sequentially increased to 75%, 85%, and 100%. After clearing, the brain tissue slab was mounted on a microscope slide, and a bright field image acquired at 2× magnification.

#### Characterization of vascular morphology

After segmenting the vasculature, we computed several morphometric parameters to characterize the 3D vascular architecture. The *fractional vascular volume* (FV) of the tumor and CL regions of interest (ROIs) was determined by computing the fractional occupancy of the binary vascular structure within each ROI. The ImageJ “3D Skeletonization” plugin was used to automatically extract the vascular centerlines or 3D skeleton [43] and compute *vessel branch lengths*. To measure *vessel radius*, a 3D Euclidean distance map [29] of the vasculature was computed using the ChamferMap module in Amira. This EDM represents the smallest distance between each vessel voxel and the background. Vessel radii were determined by multiplying the binary skeletonized image with the EDM.

#### Zonal analysis

A transition zone at the tumor-brain tissue interface approximately 200 µm wide was identified by histology based on cell density and vascular morphology. This transition zone was defined in the μMRI data from the manually drawn 3D tumor ROI. We applied 3D morphological dilation and erosion operations using a spherical structuring element (∼100 µm diameter) on the tumor ROI. The eroded ROI was subtracted from the dilated ROI to obtain the transition zone. The morphological parameters described above were calculated for the tumor ROI, transition zone and normal brain.

#### Comparison of in vivo and ex vivo MRI blood volume measurements

The *ex vivo* MRI data was co-registered to the *in vivo* MRI to compare the blood volume measurements obtained from the two techniques. The high-resolution 3D *ex vivo* MGE data was resampled in Amira (Visage Imaging Inc., CA) to match the 2D *in vivo* MRI data. Then, landmarks were manually set using the resampled *ex vivo* image and the *in vivo* pre-contrast T_2_w image. The Amira LandmarkWarp module was applied to the native *ex vivo* data. The co-registration was refined in AFNI by performing an affine registration using a least squares cost function. The same transformations applied to the *ex vivo* MGE image were applied to the segmented vessels.

#### Calculating DTI traces, fiber tracking and visualization

Voxel-wise ADC and FA maps were computed from the DWI data for each brain using DTIStudio (http://www.mrstudio.org). For each voxel, we calculated the eigenvalues of the diffusion tensor (λ_1_, λ_2_ and λ_3_), from which ADC and FA were computed according to:
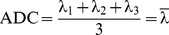
(3)

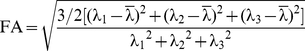
(4)3D tract reconstruction was performed using the FACT method (DTIStudio) [44], [45] with a FA threshold 0.2 and fiber angles less than 40° between two connected pixels. All fiber tract visualization and rendering of the vasculature was performed using the Neuro tool box in Amira.
